# Secondary Metabolite Profiling, Anti-Inflammatory and Hepatoprotective Activity of *Neptunia triquetra* (Vahl) Benth

**DOI:** 10.3390/molecules26237353

**Published:** 2021-12-03

**Authors:** Nasir Aziz Wagay, Shah Rafiq, Mohammad Aslam Rather, Younas Rasheed Tantray, Feng Lin, Shabir Hussain Wani, Ahmed M. El-Sabrout, Hosam O. Elansary, Eman A. Mahmoud

**Affiliations:** 1 Botany Research Laboratory, Vidya Bharati Mahavidyalya College, Amravati 444602, Maharashtra, India; nasir.wagay1989@rediffmail.com; 2 Department of Botany, Government Degree College, Baramulla 193101, Jammu and Kashmir, India; 3 Plant Tissue Culture Laboratory, Department of Botany, University of Kashmir, Srinagar 190006, Jammu and Kashmir, India; shahrafiq7@gmail.com; 4 Department of Chemistry, Government Degree College, Doda 182202, Jammu and Kashmir, India; aslam.aslam2006@rediffmail.com; 5 Plant Biotechnology Division, Indian Institute of Integrative Medicine, Sanat Nagar, Srinagar 190005, Jammu and Kashmir, India; younasrasheed53@gmail.com; 6 Department of Plant, Soil and Microbial Sciences, Michigan State University, East Lansing, MI 48824, USA; fenglin@msu.edu; 7 Mountain Research Centre for Field Crops, Sher-e-Kashmir University of Agricultural Sciences and Technology of Kashmir, Anantnag 192101, Jammu and Kashmir, India; shabirhussainwani@gmail.com; 8 Department of Applied Entomology and Zoology, Faculty of Agriculture (EL-Shatby), Alexandria University, Alexandria 21545, Egypt; elsabroutahmed@alexu.edu.eg; 9 Plant Production Department, College of Food & Agriculture Sciences, King Saud University, Riyadh 11451, Saudi Arabia; 10 Department of Food Industries, Faculty of Agriculture, Damietta University, Damietta 34511, Egypt; emanmail2005@yahoo.com

**Keywords:** anti-inflammatory, hepatoprotective, GC-HRMS, secondary metabolites

## Abstract

The present study aimed to analyze the phytoconstituents of *Neptunia triquetra* (Vahl) Benth. Anti-inflammatory and hepatoprotective activities of ethanol (EE), chloroform (CE) and dichloromethane (DCME) of stem extracts were evaluated using in vivo experimental models. The extracts were analyzed for phytoconstituents using GC-HRMS. Anti-inflammatory activity of CE, EE and DCME was accessed using carrageenan-induced paw oedema, cotton pellet-induced granuloma and the carrageenan-induced air-pouch model in Wistar albino rats. The hepatotoxicity-induced animal models were investigated for the biochemical markers in serum (AST, ALT, ALP, GGT, total lipids and total protein) and liver (total protein, total lipids, GSH and wet liver weight). In the in vivo study, animals were divided into different groups (six in each group) for accessing the anti-inflammatory and hepatoprotective activity, respectively. GC-HRMS analysis revealed the presence of 102 compounds, among which 24 were active secondary metabolites. In vivo anti-inflammatory activity of stem extracts was found in the order: indomethacin > chloroform extract (CE) > dichloromethane extract (DCME) > ethanolic extract (EE), and hepatoprotective activity of stem extracts in the order: CE > silymarin > EE > DCME. The results indicate that *N. triquetra* stem has a higher hepatoprotective effect than silymarin, however the anti-inflammatory response was in accordance with or lower than indomethacin.

## 1. Introduction

Herbal medicines are the oldest form of healthcare, and India have a long tradition of treating various disorders with these plant drugs. Simultaneously, these drugs are cheap, easily available and have minimum side effects. *Neptunia triquetra* (Vahl) Benth., commonly known as ‘Lajalu’, is one such plant that is commonly used all over India [1,2]. The plant grows as a terrestrial low prostrate perennial herb with yellow flowers. It is distributed in Maharashtra in open fields [3]. Traditionally, Kols, Gonds, Lodhs and Gujars of the Banda district in Uttar Pradesh use extract of *N. triquetra* root for dysentery [4,5]. The whole plant is a very good tonic, particularly for those who are suffering from jaundice [2]. The whole plant is astringent [6]. Stem juice is astringent and is poured into the ear to provide relief from earache. Juice of the stem is also used for curing syphilis. Juice of twigs is used to prevent gastritis, acidity and constipation. Fresh leaf juice is used as a refrigerant and also against ageing. The juice of flowers of this plant is used for eye diseases [7]. Decoction of aerial parts is used for the inflammation caused by cuts, wounds and infections [6]. The plant is edible, used as fodder, the root is astringent and the whole plant is used as an antipyretic [8]. The plant is also used for intestinal diseases [9]. Aerial parts of the plant possess moderate pancreatic lipase inhibitory activity [10].

Inflammation is a defensive response of the body, elicited by numerous stimuli such as infectious agents, environmental factors, ischemia, physiological and pathological factors, antigen–antibody reactions and free radicals. Macroscopically, erythema (redness), edema, tenderness, pain and heat are the clinical signs of the inflammatory response. Chronic inflammation involves the release of several mediators, such as interleukins 1, 2 and 3, GM-CSF (granulocyte-macrophage colony-stimulating factor), TNF-α2 (tumor necrosis factor-α2), interferon and PDGF (platelet-derived growth factor). The release of these mediators leads to rheumatoid arthritis, in which chronic inflammation results in pain and destruction of bone and cartilage [11,12]. Despite the discovery of cortisone and the emergence of several newer agents, the search for better anti-inflammatory drugs continues because these agents have many side effects as well as very high potency, and none of them are suitable for prolonged use.

The liver has paramount importance in the excretion and metabolism of an organism. The task of neutralization of chemotherapeutic agents, environmental pollutants and xenobiotics is constantly carried out by the liver. Thus, many and varied diseases are associated with this organ, however chemicals damage the mitochondria of the liver cells, which is generally known as hepatotoxicity. The abnormal functioning of the liver aids in excessive release of oxidants and enzyme activation in the Cyt P-450 system (such as CYP2E1), resulting in injuries to the liver cells and also leading to oxidative stress [13]. Injury to hepatocyte and bile duct cells leads to the accumulation of bile acid inside the liver [14]. Leukocytes (i.e., monocytes and neutrophils), fat-storing stellate cells and Kupffer cells also have a role in the mechanism. Liver damage is often characterized by biochemical markers such as *alkaline phosphatase* (ALP), *alanine transferase* (ALT) and bilirubin. Liver damage is further characterized by initial *alanine transferase* elevation (hepatocellular type) and initial *alkaline phosphatase* rise (cholestatic type). However, they are not mutually exclusive and mixed types of injuries are often encountered [15,16]. Symptomatic relief is caused by immunosuppressive and corticosteroid agents, however the search for more efficient curative drugs is necessary [17]. The present study emphasizes the evaluation of active compounds present in the *N. triquetra* and its biological activities.

## 2. Results

### 2.1. Phytochemical Studies of N. triquetra (Roots, Stem and Leaves)

#### 2.1.1. Analytical/Physiochemical Analysis

The percentage values suggest that moisture content was higher in leaves, followed by roots and stem. The percentage of ash yield was higher in the stem, closely followed by roots and leaves. The ash of the stem showed the most dissolution in acid, while the ash of roots and the stem was almost equally dissolvable in water. The extractive values were found to be higher in the case of ethanol as a solvent, followed by chloroform. The least extractive values were those of the solvent dichloromethane (Table 1).

#### 2.1.2. Powder Study

The characteristic analyses of the dried powder of various parts of the plant under study are depicted in Table 2, Table 3, Table 4 and Table 5.

#### 2.1.3. Qualitative Phytochemical Analysis

The qualitative phytochemical screening of *N. triquetra* in four extracts, i.e., water, dichloromethane, ethanol and chloroform, showed that there was a prominent presence of phytoconstituents such as alkaloids, flavonoids, phenols, saponins, steroids, tannins and terpenoids. However, glycosides were not found in *N. triquetra* part extracts. The phytoconstituents were detected in most of the extracts (Table 6).

#### 2.1.4. Chromatographic Studies

The chromatograms and identified compounds with their retention time, approximate concentration in the extract (peak area %), molecular weight, molecular formula and structures of identified secondary metabolites are presented in Supplementary File S1. GC-HRMS results of *N. triquetra* roots extracted in chloroform, dichloromethane and ethanol resulted in the identification of 13 compounds (Supplementary Figure S1 and Table S1), 12 compounds (Supplementary Figure S2 and Table S2) and 12 compounds (Supplementary Figure S3 and Table S3), respectively. Similarly, GC-HRMS results of *N. triquetra* stem in chloroform (Supplementary Figure S4 and Table S4), dichloromethane (Supplementary Figure S5 and Table S5) and ethanol (Supplementary Figure S6 and Table S6) resulted in **8** and **13** compounds, respectively. GC-HRMS results of *N. triquetra* leaves in chloroform (Supplementary Figure S7 and Table S7), dichloromethane (Supplementary Figure S8 and Table S8) and ethanol (Supplementary Figure S9 and Table S9) resulted in **13**, **14** and **9** compounds, respectively. A total of 102 compounds were identified by GC-HRMS in *N. triquetra,* among which 1 is a flavonoid, 2 are alkaloids, 1 is a glucosinolate, 7 are terpenoids, 3 are phytosterols, 1 is a secosteroid, 5 are phenols, 1 is a lactone, 1 is a ketone derivative, 1 is a napthoquinone derivative and 1 is a heterocyclic compound. The secondary metabolite profile of *N. triquetra* identified by GC-HRMS is presented in Table 7.

### 2.2. Biological Activity

Most of the compounds were identified in the stem extracts; hence, they were further studied for their anti-inflammatory and hepatoprotective activities.

#### 2.2.1. Anti-Inflammatory Activity of *N. triquetra*

The ethanolic extract (EE), chloroform extract (CE) and dichloromethane extract (DCME) of *N. triquetra* were evaluated for anti-inflammatory activity in acute and chronic experimental animal models, and the results are summarized in Table 8, Table 9 and Table 10.

##### Effect on Carrageenan-Induced Rat Paw Edema

The results for the effect of *N. triquetra* on carrageenan-induced rat paw edema have been presented in Table 8, and it is evident that CE showed maximum inhibition in the edema induced by carrageenan, followed by EE and DCME. The CE at 50, 100 and 200 mg/kg doses inhibited the edema by 48.65%, 55.41% and 55.41% respectively, after 3 h, whereas the standard drug showed 55.41% inhibition after 3 h, as compared to the control group. The EE extract at 50, 100 and 200 mg/kg doses showed 29.73%, 37.84% and 43.24% inhibition in edema after 3 h, respectively. The DCME showed the least reduction in edema. These results indicate that the CE possesses the strongest anti-inflammatory activity as compared to EE and DCME at every hour after pretreatment. The effects of CE at 100 and 200 mg/kg body weight at 2 and 3 h were comparable to that of the standard drug indomethacin. The pretreatment with EE, CE and DCME resulted in a significant and dose-dependent reduction in carrageenan-induced paw edema in rats.

##### Effect on Cotton Pellet Granuloma in Rats

The effect of EE, CE and DCME on granuloma formation in the cotton pellet method is presented in Table 9, which shows that the inhibition caused by CE at the dose of 200 mg/kg was the maximum (50.47%) and was comparable with that of the standard drug indomethacin, which caused 56.29% inhibition, as compared to the control. The EE and DCME showed almost the same effect. However, the effect of all the extracts was increased with the increase in dose and the maximum was found at a dose of 200 mg/kg. The order of inhibition was found to be indomethacin > CE > DCME > EE.

##### Effect on Exudate Volume, Neutrophil and Monocyte Count in Carrageenan-Induced Air-Pouch Inflammation

The results of EE, CE and DCME on carrageenan-induced air pouch in rats are shown in Table 10, which shows that each extract significantly (*p* < 0.05) reduced the exudate volume and infiltration of neutrophils and monocytes into the air pouch compared to the control group. The reduction was found to be dose-dependent. The CE showed a significant effect on these parameters at a lower dose than EE and DCME when compared to the standard group. The order of activity was found to be indomethacin > CE > DCME > EE. The results of the study in carrageenan-induced air-pouch inflammation suggest that the EE, CE and DCME in different doses significantly suppressed carrageenan-induced exudate volume and neutrophil and monocyte count in rats, and demonstrated significant anti-inflammatory activity.

#### 2.2.2. Hepatoprotective Activity of *N. triquetra*

##### Effect on Biochemical Parameters

Changes in the activities of marker enzymes (AST, ALT, ALP and GGT), total lipids and total protein content in the serum of CCl_4_-induced liver damage in rats by *N. triquetra* are presented in Table 11. The levels of serum marker enzymes AST, ALT, ALP, GGT and total lipids were found to be significantly increased, whereas the protein content was significantly decreased in CCl_4_-induced liver damage rats (Group II) when compared with the normal group (*p <* 0.05). A significant decrease in AST, ALT, ALP, GGT and total lipids, and an increase in total protein were observed in the serum of rats treated with EE, CE and DCME at the doses of 50, 100 and 200 mg/kg body weight, as compared to that of the control group (Group II). A decrease in serum enzymes and total lipids and an increase in the total protein were also found in the group treated with silymarin (Group III, 25 mg/kg). The total protein contents of the liver were found to be low in CCl_4_-treated animals (Group II), and attained an almost normal value in the rats treated with silymarin, EE, CE and DCME. The total lipid concentration of the liver was high in Group II animals, while it attained an almost normal value in the animals treated with silymarin, EE, CE and DCME (Table 11). From Table 11, it is evident that the hepatoprotective effect offered by CE was found to be greater than that by EE and DCME. A significant reduction in wet liver weight was observed in the animals treated with silymarin and plant extracts, and an increase in the wet liver weight was observed in the CCl_4_-treated group as compared with the control.

##### Effect on GSH Level in Liver Tissues

The effect of EE, CE and DCME on glutathione content in the liver is shown in Table 12. The GSH level of liver homogenate in the CCl_4_ control group (0.78 ± 0.05 µmol/g of the liver) was found to be significantly lower than that in the normal group (5.63 ± 0.03 µmol/g of the liver). The GSH level of animals treated with EE, CE and DCME was found to be greater than that of the CCl_4_-treated group. The value of GSH in the rats treated with EE, CE and DCME at 200 mg/kg was found to be 5.25 ± 0.08, 5.43 ± 0.08 and 3.93 ± 0.11 µmol/g of liver, respectively. The GSH level in CE (at every indicated dose) and EE (200 mg/kg) treated groups was measured to be higher than that in the CCl_4_ control group (*p* < 0.05). The results indicated that silymarin, CE and EE almost completely restored the glutathione level in CCl_4_-treated groups to the normal level.

**Figure 1:**
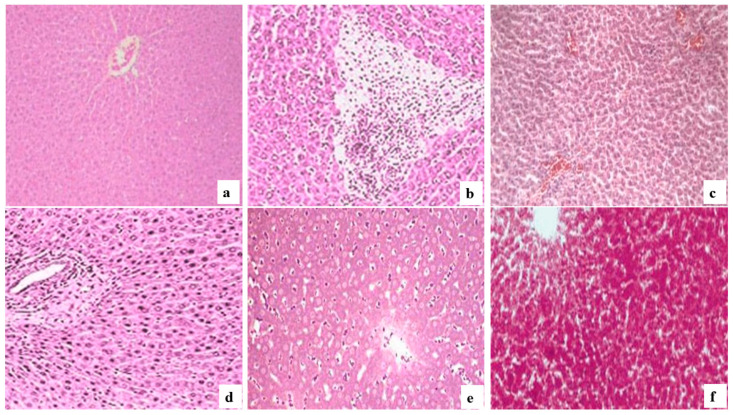
Photomicrographs of Architecture of hepatic cells during Hepatoprotective activity. (**a**) Photomicrograph of Liver section from Control group (LP only) rats showing normal hepatic architecture. (**b**) Photomicrograph of Liver section from Carbon tetrachloride treated rats showing severe hepatotoxicity. (**c**) Photomicrograph of Liver section from Silymarin treated rats showing almost normal architecture. (**d**) Photomicrograph of Liver section from DCME (200 mg/kg) treated rats showing almost normal architecture. (**e**) Photomicrograph of Liver section from EE (100 mg/kg) treated rats showing almost normal architecture. (**f**) Photomicrograph of Liver section from CE (50 mg/kg) treated rats showing almost normal architecture.

The hepatoprotective activity of the extracts was in the order of CE > silymarin > EE > DCME. The chloroform extract of *N. triquetra* showed the strongest effect; therefore, it can be concluded that the hepatoprotective effect lies in the chloroform fraction (Figure 1).

## 3. Discussion

Recently, vast development in the synthetic drug discovery field has been achieved, however, it has been found sooner or later that every synthetic drug has its side effects, and this property of synthetic drugs urges the need for drugs with minimum or no side effects, hence making us rely on the medicinal plants and their chemical constituents. *N. triquetra* is traditionally used for curing various diseases, such as jaundice, syphilis, inflammatory diseases and intestinal diseases, in the folk medicine of India.

Analytical analysis or physiochemical parameters, such as moisture content, ash values and extractive values, of any plant species in different solvents are considered as indicators of chemical constituents for the species. Ash value is used to determine the quality and purity of a crude drug; however, ash simply represents inorganic salts naturally occurring in a drug or the amount of a chemical element remaining after ignition. The constituent nature of the crude drug is also evaluated by the extractive values; however, the variability in these parameters might be due to certain climatic or geographic changes. An organoleptic character plays an important role in the identification of crude drugs, while the fluorescent analysis relates the reactivity of plant powders (crude drug) with different chemical reagents. Qualitative analysis and GC-HRMS analysis confirmed the prominent presence of secondary metabolites in *N. triquetra* (Table 6 and Table 7).

Among the identified secondary metabolite compounds, 3-butylindolizidine is an indolizidine alkaloid, and this group have resemblances with the structure and pharmacological effects of anti-inflammatory drugs such as indomethacin [18] and have been previously reported in plant extracts [19,20]. Therapeutically, terpenoids also have a wide spectrum of activities [21] and could ameliorate various symptoms caused by inflammation [22]. In *N. triquetra* extracts, terpenoids with reported anti-inflammatory activities are α-caryophyllene or humulene [23,24,25], phytol [26,27] and lupeol [28,29]. Terpenoids in *N. triquetra* extracts with reported antioxidant and hepatoprotective activity are squalene [28,30] and lupeol [31]. Cycloartenol acetate is a triterpenoid and precursor molecule of phytosterols [32] and has also been reported to possess anti-inflammatory activity [33]. α-Amyrin is also a triterpene possessing anti-inflammatory [34] and hepatoprotective activity [35,36]. Dihydrogeraniol is a monoterpenoid, mostly found in essential oils, and has been reported for its various biological activities. An identified flavonoid having anti-inflammatory activity in *N. triquetra* extracts is scandenone (or warangalone) [37]. Phenolics are the most diverse group of secondary metabolites and mostly act as antioxidants, i.e., they are able to scavenge free radicals. Phenol-2,4-bis(1,1-dimethylethyl) has been reported for anti-inflammatory activity [38] as well as antioxidant activity [24,39], and other identified phenolic compounds with antioxidant activity are phenol-2,6-dimethoxy [40] and pyrogallol [41]. Pyrogallol has prominent hepatoprotective activity [42]. 4-Hydroxy-2-methylacetophenone (or paeonol) [43] also has hepatoprotective activity [44]. 4-Propoxyphenol has been reported in plants [45,46], however it has not been evaluated for anti-inflammatory or hepatoprotective activity. Stigmasterol, γ-sitosterol and campesterol are the identified phytosterols and have been reported for their anti-inflammatory [47,48,49,50,51,52,53] and antioxidant activities [54,55,56]. 24,25-Dihydroxy vitamin D is a biologically active secosteroid. Among the other identified compounds, some that have already been reported for possessing anti-inflammatory activity are 4*H*-pyran-4-one, 2,3-dihydro-3,5-dihydroxy-6-methyl [57,58,59], benzofuran, 2,3-dihydro [60,61], 2(4*H*)-benzofuranone and 5,6,7,7a-terahydro-4,4,7a-trimethyl-(*R*) [24], however,3,3′-dimethyl-1′-hydroxy-5,8-dimethoxy-2,2′-binapthalene-1,4,5′,8′-tetrone, desulphosinigrin and 1-butylpyrrolidine have not been evaluated for said biological activity until now (Table 6 and Table 7). 

The efficacy of medicinal plants against inflammation or as herbal anti-inflammatory agents should be achieved by a systematic approach. The major components that induce pain and inflammation are prostaglandins and leukotrienes [62,63]. Paw oedema induction by carrageenan is due to the involvement of various inflammatory mediators [64]. Oral administration of doses of CE at 200 mg/kg showed 56.26% inhibition in the case of carrageenan-induced rat paw edema and 49.87% inhibition in cotton pellet granuloma in rats. EE and DCME also showed significant anti-inflammatory activity in carrageenan-induced rat paw edema and cotton pellet-induced granuloma, confirming the effectiveness of all three extracts in chronic inflammatory conditions. The inflammation repair process involves the formation of granulation tissue, which is a reddish mass with high vascularization, and this tissue is formed by continuous divisions of fibroblasts, macrophages, neutrophils and small blood vessel multiplications [65,66]. The carrageenan-induced air-pouch model was selected to assess the efficacy of the extract against the proliferative phase of inflammation. In this model, the EE, CE and DCME significantly reduced infiltration of neutrophils and monocytes (Table 10). Enzymes secreted by the lysosomes play a major role in the development of chronic and acute inflammation [67,68,69,70], and most of the anti-inflammatory drugs exert their beneficial effects by inhibiting either the release of these lysosomal enzymes or by stabilizing the lysosomal membrane at the site of inflammation [71].

The liver, with its metabolic and detoxifying abilities, plays a vital role [72]. When the liver is subjected to a variety of endogenous and xenobiotic substances, it produces a variety of intermediate and end products that can induce hepatocellular death and are the main causes of liver disease [73]. Traditional therapy focuses on symptom control and liver transplantation in severe cases of liver disease to ensure an individual’s survival and maintain liver function [74]. However, no medications are currently available to boost the organ’s detoxifying capacity. Chemical-induced hepatotoxicity is a critical issue all over the world, and with this perspective the efficacy of plant extracts is being tested for the generation of hepatoprotective medicines. The most effective and extensively used criteria for assessing the hepatoprotective efficacy of plant extracts is its comparison with CCl_4_, a powerful hepatotoxic toxin [75]. Mice administered with CCl_4_ developed infiltration, vacuolization, and inflammation in the liver, resulting in increased liver weight and a reduction in body weight [76] (Figure 1b). In contrast to the negative control, mice pre- and post-treated with EE, CE and DCME revealed no significant differences in body weight, absolute and relative liver weight. In the present study, EE, CE and DCME seemed to offer protection and maintain the structural integrity of hepatic cells. CE was more effective than EE and DCME for AST (21.83 IU/L), ALT (34.36 IU/L), ALP (76.81 IU/L) and GGT (4.30 IU/L), even at 50 mg/kg. The CE of *N. triquetra* showed the strongest effect, and therefore it can be concluded that the hepatoprotective effect lies in the chloroform fraction. The EE, CE and DCME reduced the levels of biochemical parameters in serum in a dose-dependent manner: the lower dose had a lesser impact on the indicators of liver damage, whereas the medium and higher doses were able to significantly reduce the levels of AST, ALT, ALP, GGT, total protein and total lipids in serum (Table 11). This might indicate that the lower dose is below the minimum effective dose, which cannot induce a substantial reduction in liver enzyme levels, while the other two doses are high enough to cause a considerable reduction in liver enzyme levels (Table 11). The hepatoprotective activity of the extracts was in the order: CE > silymarin > EE > DCME. Carbon tetrachloride impairs the capacity of the liver to synthesize albumin, which in turn decreases the protein content in serum [77,78]. The retrieval of protein concentration to normalcy further confirms the hepatoprotective effect of *N. triquetra*. Additionally, the GSH level of animals treated with EE, CE and DCME was found to be higher than CCl_4_-induced rats and was found to be higher (EE and CE at 200 mg/kg) or almost equivalent (CE at 100 mg/kg) to silymarin-treated rats, which is further evidence of the hepatoprotective effect of *N. triquetra*.

## 4. Materials and Methods

### 4.1. Collection and Identification of Plant Material

The *N. triquetra* was collected during all growing periods (2015–2017) from Chikhalgaon village, Patur Taluka, Akola, Maharashtra, India (20°45′7228″ N and 76°93′0060″ E). The plant grows as a low prostrate perennial herb, with stems ascending, slender, compressed and more or less angular. Branches are procumbent or ascending, and leaves are abruptly bipinnate, acuminate and persistent, with pinnae having 2–3 pairs, and shortly stalked. Leaflets have 10–15 pairs, are sessile, 3–6 by 2 mm and glabrous. Flowers are yellow (the sterile flowers are few or absent) in globose heads, peduncles are solitary, axillary and slender, with 1 or 2 large ovate distant bracts on the peduncle. Flowers and fruiting occur during August–October. The plant species are also treated under various synonyms: *Desmanthus triquetra* Wild., *Mimosa triquetra* Vahl ex Roxb. and *M. natans* Linn. Morphologically, collected specimens were identified with the help of standard floras and authenticated by a taxonomist. The collected specimens were also compared with the specimens lying in the BSI and Central National Herbarium, Calcutta. Voucher specimens (NAW/1264-12/08/2015) were deposited in the Herbarium House, Department of Botany, Vidyabharati Mahavidyalaya College, Amravati, Maharashtra, India.

### 4.2. Preparation of Extracts

The Soxhlet extraction technique was used for extraction from dried plant parts in three solvents: chloroform, dichloromethane and ethanol. After extraction in the Soxhlet apparatus for 24 h, the extracts (CE, DCME and EE, respectively) were filtered, concentrated using a rotatory vacuum evaporator and were subjected to further analysis. The phytochemical analysis was carried out separately for root, stem and leaf extracts, and anti-inflammatory and hepatoprotective activity were studied for the stem extracts.

### 4.3. Phytochemical Studies

#### 4.3.1. Analytical Methods

The Indian Pharmacopoeia procedures [79] and recommended procedures [80] were followed for physiochemical or analytical studies. Moisture percentage, total ash values, acid-insoluble ash and acid-soluble ash, water-soluble and water-insoluble ash and extractive values (in chloroform, ethanol and dichloromethane) for parts of the plant under study were found.

#### 4.3.2. Powder Study

Organoleptic evaluations (color, odor, taste surface characteristics) of dried powder were carried out by the standard procedure of the Indian Pharmacopoeia [79]. The fluorescent behavior analysis for each part of plant under study was carried out by using standard methods of Pratt and Chase [81].

#### 4.3.3. Qualitative Phytochemical Analysis

The extracts were prepared in the solvents water, dichloromethane, ethanol and chloroform by the Soxhlet method, in which 10 g of powder was extracted in 180 mL of solvent by maintaining the temperature at the boiling point of the solvent for 3–18 h [82].

The qualitative analysis for secondary metabolites of all plant parts under study was performed by using standard methods [83]. For detecting alkaloids, Wagner’s reagent and Mayer’s reagent tests were applied. Flavonoids were detected using the sodium hydroxide test and the lead acetate test. Glycosides were detected by using the Killer–Killani test and Fehling’s test. The phenol compounds were detected by the phenols test. Saponins’ detection was performed using a froth and foam test. Steroids were detected by using the Salkowski test and Libbermann–Burchard’s test. Tannin compounds were detected using the ferric chloride test. Terpenoids were detected using the Salkowski test.

#### 4.3.4. Gas Chromatography–High-Resolution Mass Spectrometric Analysis

The analysis of samples was carried out using GC-HRMS at the Sophisticated Analytical Instrument Facility, IIT Powai, Mumbai, Maharashtra, India. The GC chromatogram displays retention times, while MS analysis involves the fragmentation pattern of compounds, mass peak, base peak, *m/z* values, peak intensities, etc. Along with these *m/z* values, a matching number of peaks were used to confirm the compound identification. The information acquired through this was the name, structure, molecular weight, molecular formula and relative quantity of compounds. The online database which aided in the identification of compounds was METLIN. The metabolite mass spectral database, National Institute of Standard and Technology (NIST) Database, Mass Bank, Respect for Phytochemical and Golm metabolome databases were also used. The structures of identified metabolites were drawn by the software ChemAxon (MarvinSketch 16.3.21.0, Cambridge, MA, USA).

### 4.4. Biological Activity

#### 4.4.1. Determination of Anti-Inflammatory Activity of *N. triquetra*

Healthy Albino rats (Wistar strain), weighing between 100 and 160 g, of either sex were selected and provided rat feed and water ad libitum. The animals had free access to food and water and were maintained under a controlled temperature (27 ± 2 °C) and a 12/12 h light/dark cycle. Records of the initial body weight of every animal were maintained. The carrageenan-induced paw edema model (acute model) [84], carrageenan-induced air-pouch model (Subacute model) [85,86] and cotton pellet-induced granuloma model [84] were used.

##### Carrageenan–Induced Rat Paw Edema Method

Eleven groups were formed with six animals (*n* = 6) in each group. The first and second groups received acacia, i.e., the vehicle only (5%, 10 mL/kg), and indomethacin (10 mg/kg) respectively, and served as control and standard groups, respectively. The third, fourth and fifth groups received EE orally at 50, 100 and 200 mg/kg doses, respectively. The sixth, seventh and eighth groups received CE orally at 50, 100 and 200 mg/kg doses respectively, and the ninth, tenth and eleventh groups received DCME orally at 50, 100 and 200 mg/kg doses, respectively.

The drugs were administered orally with the help of an oral catheter half an hour before the carrageenan suspension administration, the 0.1 mL of carrageenan suspension (1%, i.e., 10 mg/mL) prepared in 5% acacia was injected in the left hind paw (sub plantar region) of each rat.

The plethysmometer was used to measure the paw volume immediately after injection (i.e., 0.0 h) as well as after 1, 2, 3 and 4 h. The average paw swelling in the group of extract-treated rats was compared with the control group and the standard group.

The formula used to calculate the percentage change in edema was:

(1) $$\mathrm{Percent}\;\mathrm{edema}\;\mathrm{inhibition}=1\;-\;\frac{\mathrm{Volume}\;\mathrm{of}\;\mathrm{treated}\;\mathrm{group}}{\mathrm{Volume}\;\mathrm{of}\;\mathrm{control}\;\mathrm{group}}\;\times \;100$$

##### Cotton Pellet-Induced Granuloma

The rats were divided into different groups (*n* = 6), as discussed under the acute model. Light ether was used to anaesthetize the rats which were incised by blunted forceps on the lumbar region, and a sterilized cotton pellet (100 ± 1 mg) was inserted in the subcutaneous tunnel made in the groin area. All the animals received either EE/CE/DCME, indomethacin or vehicle (1% CMC) orally, depending upon their respective grouping, for seven consecutive days from the day of cotton pellet insertion. The rats were anaesthetized again on the eighth day and the cotton pellets were removed, which were then dried to a constant mass [84].

##### Carrageenan-Induced Air-Pouch Model

The rats were divided into different groups (*n* = 6), as discussed under the acute model. The method of Salvemini et al. [87] was followed to produce the air pouch. After anesthetization of rats, 20 mL of sterile air was subcutaneously injected into the intra-scapular area of the back (i.e., 0 days) to form the air cavities. To keep the space open in the cavity, an additional 10 mL sterile air was injected every third day (i.e., third and sixth days). An inflammatory response was induced by directly injecting 2 mL of a 1% solution of carrageenan dissolved in saline into the pouch on the seventh day. The rats were orally pre-treated with either vehicle, EE/CE/DCME or indomethacin 2 h before the injection of carrageenan. After 24 h of the first treatment, the second dose was repeated. The rats were anaesthetized with ether after 48 h of carrageenan injection and the pouch was opened carefully by making a small incision. The exudate volume was collected and measured. Differential cell counts (monocytes and neutrophils) were counted by a manual cell counter in the aliquot of collected exudates after staining with the Wright’s stain.

#### 4.4.2. Determination of Hepatoprotective Activity of *N. triquetra*

The procedure, technique and biochemical estimations were carried out by using the method of Venukumar and Latha [88]. The CCl_4_-induced hepatoprotective model was used in the present study.

Male Albino rats weighing between 100 and 120 g were fed on a standard pellet diet and water ad libitum. The rats were allowed a one-week acclimatization period before experimental sessions. An acute toxicity study was performed as per OECD-423 guidelines and then the doses were selected accordingly [89].

##### Administration and Dosage Preparation for Testing

Liver damage was induced in rats by administering CCl_4_ subcutaneously (SC) in the lower abdomen in a suspension of liquid paraffin (LP) in the ratio 1:2 *v/v* at the dose of 1 mL CCl_4_/kg body weight of each animal.

##### Experimental Design

The animals were divided into twelve groups of six animals each, as follows:

1. Group I (Positive Control): Animals served as a control group and received SC administration of LP only at the dose of 3 mL/kg body weight, on alternate days for a duration of 14 days.

2. Group II (Negative Control): Animals were treated with SC administration of LP + CCl_4_ on alternate days for a duration of 14 days.

3. Group III (Standard): Animals were treated with SC administration of LP + CCl_4_ on alternate days and silymarin orally at the dose of 50 mg/kg body weight daily for 14 days.

4. Group IV: Animals were treated with SC administration of LP + CCl_4_ on alternate days and EE suspension orally at the dose of 50 mg/kg body weight daily for 14 days.

5. Group V: Animals were treated with SC administration of LP + CCl_4_ on alternate days and EE suspension orally at the dose of 100 mg/kg body weight daily for 14 days.

6. Group VI: Animals were treated with SC administration of LP + CCl_4_ on alternate days and EE suspension orally at the dose of 200 mg/kg body weight daily for 14 days.

7. Group VII: Animals were treated with SC administration of LP + CCl_4_ on alternate days and CE suspension orally at the dose of 50 mg/kg body weight daily for 14 days.

8. Group VIII: Animals were treated with SC administration of LP + CCl_4_ on alternate days and CE suspension orally at the dose of 100 mg/kg body weight daily for 14 days.

9. Group IX: Animals were treated with SC administration of LP + CCl_4_ on alternate days and CE suspension orally at the dose of 200 mg/kg body weight daily for 14 days.

10. Group X: Animals were treated with SC administration of LP + CCl_4_ on alternate days and DCME suspension orally at the dose of 50 mg/kg body weight daily for 14 days.

11. Group XI: Animals were treated with SC administration of LP + CCl_4_ on alternate days and DCME suspension orally at the dose of 100 mg/kg body weight daily for 14 days.

12. Group XII: Animals were treated with SC administration of LP + CCl_4_ on alternate days and DCME suspension orally at the dose of 200 mg/kg body weight daily for 14 days.

On the fifteenth day, the animals were sacrificed by decapitation by making an incision on the jugular vein to collect blood. The liver tissue was dissected out and blood was blotted off, then it was washed in saline and weighed instantaneously to obtain the wet weight.

##### Biochemical Parameters

Serum was separated from the collected blood and subjected to biochemical estimations of different parameters, such as *gamma glutamyl trans-peptidase* (GGT) [90], *aspartate aminotransferase* (AST) [91], *alkaline phosphatase* (ALP) [92] and *alanine aminotransferase* (ALT) [91]. Biochemical estimations such as total lipids [93] and total proteins [94] of liver homogenates were also calculated.

##### Glutathione Estimation

The estimation of glutathione (GSH) was performed using the procedure described by Ellman [95]. The tissue proteins were precipitated by the addition of 20% trichloroacetic acid containing 1 mM of EDTA, with an equal volume of tissue homogenates dissolved in 0.1 M of phosphate buffer (pH 7.4). The mixture was centrifuged at 200 rpm for 10 min and the supernatant (200 µL) was then transferred to a new set of test tubes and 1.8 mL of Ellman’s reagent was added. The volume of the mixture in each test tube was made up to 2 mL and absorbance was recorded at 412 nm against a blank [96].

### 4.5. Data Analysis

The data are expressed as a mean ± standard error (SE) of the mean. Results were analyzed using one-way ANOVA followed by Tukey’s test. Differences were considered as statistically significant at *p* < 0.05, when compared to the control, using SPSS ver. 23 (SPSS Inc., Chicago, IL, USA).

## 5. Conclusions

The extracts were analyzed for phytochemical analysis (analytical, powder, qualitative, quantitative, GC-HRMS). This study was intended to demystify the biological activities such as anti-inflammatory and hepatoprotective effects of *N. triquetra*. The results showed that *N. triquetra* extracts have potent biological activity, and the reason for this activity is the diverse variety of phytochemicals identified by the phytochemical analysis. These phytochemicals can be used as an effective remedy for various ailments and drug formulations in the future, either alone or in combination with other suitable agents. There were no previous reports on these biological activities and phytochemical analysis for *N. triquetra*. Therefore, this study will form the foundation of this plant, and further pharmacological studies with proper clinical trials are suggested.

## Figures and Tables

**Table 1:** Analytical values of *N. triquetra*.

S. No.	Parameter Studied		Percentage Value (*w*/*w*)		
			Roots	Stem	Leaves
1.	Moisture Content		5.13 ± 0.49	3.79 ± 0.51	7.28 ± 0.56
2.	Total Ash		5.85 ± 0.72	6.67 ± 0.61	4.21 ± 0.03
3.	Acid-soluble Ash		10.74 ± 0.43	13.95 ± 0.35	11.99 ± 1.63
4.	Acid-insoluble Ash		88.27 ± 0.97	85.85 ± 0.64	87.94 ± 1.19
5.	Water-soluble Ash		15.27 ± 1.87	15.79 ± 0.50	12.59 ± 0.45
6.	Water-insoluble Ash		79.5 ± 0.79	80.83 ± 1.11	88.05 ± 1.18
7.	**Extractive Values in**	Chloroform	8.17 ± 0.25	6.82 ± 0.28	4.00 ± 0.41
		Ethanol	12.63 ± 0.61	10.57 ± 0.48	6.87 ± 0.43
		Dichloromethane	3.26 ± 0.71	2.85 ± 0.39	2.73 ± 0.24

The data represent mean value ± SE (standard error), *n* = 3, where *n* = no. of repetitions.

**Table 2:** Organoleptic evaluation of *N. triquetra*.

S. No.	Particulars	Observation		
		Root	Stem	Leaf
1.	Color of powder	Grayish black	Grayish green	Green
2.	Odor	Light sweet	Odorless	Odorless
3.	Taste	Tasteless	Tasteless	Tasteless
4.	Texture	Smooth	Rough	Smooth

**Table 3:** Fluorescent behavior of *N. triquetra* root powder upon treatment with different chemical reagents.

S. No.	Powder + Reagent Used	Roots	
		Visible Light	UV Light
1.	Powder as such	Light brown soil color	Light brownish
2.	Powder + Conc. H_2_SO_4_	Black	Black
3.	Powder + Conc. HNO_3_	Orange red	Yellow
4.	Powder + Conc. HCl	Brown	Light brown
5.	Powder + 10% NaOH	Reddish dark brown	Dark blackish brown
6.	Powder + 1 N HCl	Transparent yellow	Green
7.	Powder + Iodine solution	Fluorescent brown purple	Purple (light bluish)
8.	Powder + 5% FeCl_3_	Light yellow	Light greenish fluorescent
9.	Powder + KI	Cream white	Cream transparent
10.	Powder + 1 N HNO_3_	Cream white	Cream transparent
11.	Powder +1 N H_2_SO_4_	Yellowish transparent	Light yellowish transparent
12.	Powder + Ethyl acetate	Light yellow	Green

**Table 4:** Fluorescent behavior of *N. triquetra* stem powder upon treatment with different chemical reagents.

S. No.	Powder + Reagent Used	Stem	
		Visible Light	UV Light
1.	Powder as such	Grey	Whitish grey
2.	Powder + Conc. H_2_SO_4_	Black	Black
3.	Powder + Conc. HNO_3_	Orange red	Yellow
4.	Powder + Conc. HCl	Light brown	Green
5.	Powder + 10% NaOH	Dark blackish brown	Black brownish
6.	Powder + 1 N HCl	Light brown	Green
7.	Powder + Iodine solution	Transparent soil color	Purple transparent
8.	Powder + 5% FeCl_3_	Light yellowish transparent	Greenish transparent
9.	Powder + KI	Transparent	Transparent
10.	Powder + 1 N HNO_3_	Fluorescent green	Light green
11.	Powder + 1 N H_2_SO_4_	Yellowish transparent	Transparent
12.	Powder + Ethyl acetate	Dark brown	Blackish brown

**Table 5:** Fluorescent behavior of *N. triquetra* leaf powder upon treatment with different chemical reagents.

S. No.	Powder + Reagent Used	Leaves	
		Visible Light	UV Light
1.	Powder as such	Green	Green
2.	Powder + Conc. H_2_SO_4_	Black	Black
3.	Powder + Conc. HNO_3_	Orange	Orange
4.	Powder + Conc. HCl	Light green	Green
5.	Powder + 10% NaOH	Black	Black
6.	Powder + 1 N HCl	Light brown	Green
7.	Powder + Iodine solution	Soil transparent	Greenish transparent
8.	Powder + 5% FeCl_3_	Yellowish transparent	Transparent
9.	Powder + KI	Transparent	Transparent
10.	Powder + 1 N HNO_3_	Green	Green
11.	Powder + 1 N H_2_SO_4_	Transparent water	Transparent watery
12.	Powder + Ethyl acetate	Light brown	Green

**Table 6:** Qualitative phytochemical screening of *N. triquetra*.

S. No.	Constituents	Chemical Tests	ROOT				STEM				LEAVES			
			W	D	E	C	W	D	E	C	W	D	E	C
**1.**	**Alkaloids**	Wagner’s test	**+ +**	**+ +**	**+ +**	**+ −**	**+ +**	**+ −**	**+ +**	**+ +**	**+ +**	**+ +**	**+ +**	**+ +**
		Mayer’s test	**+ −**	**+ +**	**+ -**	**− −**	**+ +**	**− −**	**+ +**	**+ −**	**+ −**	**+ +**	**+ −**	**+ −**
**2.**	**Flavonoids**	Sodium hydroxide test	**+ +**	**+ −**	**+ +**	**++**	**+ −**	**+ +**	**+ +**	**+ +**	**+ +**	**+ +**	**− −**	**+ +**
		Lead acetate test	**+ +**	**− −**	**+ +**	**++**	**− −**	**+ +**	**+ +**	**+ +**	**+ +**	**+ +**	**+ +**	**+ +**
**3.**	**Glycosides**	Killer–Killiani test	**− −**	**− −**	**− −**	**− −**	**− −**	**− −**	**− −**	**− −**	**− −**	**− −**	**− −**	**− −**
		Fehling’s test	**− −**	**+ −**	**− −**	**− −**	**− −**	**− −**	**− −**	**− −**	**− −**	**− −**	**+ +**	**− −**
**4.**	**Phenols**	Phenol test	**− −**	**+ −**	**− −**	**++**	**− −**	**+ +**	**+ +**	**+ -**	**− −**	**− −**	**+ +**	**++**
**5.**	**Saponins**	Frothing/Foam test	**+ +**	**− −**	**+ +**	**− −**	**+ +**	**− −**	**+ +**	**++**	**+ +**	**− −**	**+ +**	**++**
**6.**	**Steroids**	Salkowski’s test	**− −**	**− −**	**− −**	**+ +**	**+ -**	**− −**	**+ +**	**++**	**+ +**	**− −**	**+ +**	**++**
		LB test	**− −**	**+ −**	**+ +**	**− −**	**+ -**	**− −**	**+ +**	**++**	**+ +**	**− −**	**− −**	**++**
**7.**	**Tannin**	Ferric chloride test	**+ +**	**+ −**	**+ +**	**++**	**+ +**	**+ +**	**+ +**	**++**	**+ +**	**− −**	**+ +**	**− −**
**8.**	**Terpenoids**	Salkowski’s test	**− −**	**− −**	**− −**	**++**	**+ +**	**+ +**	**+ +**	**+ +**	**− −**	**− −**	**+ +**	**++**

Note: ‘+’ = present and ‘−’ = absent. W = water extract; D = dichloromethane extract; E = ethanol extract; C = chloroform extract.

**Table 7:** Secondary metabolite profile of *N. triquetra* identified by GC-HRMS.

S. No.	Name of Identified Compound	Category	Found in Part/Parts
1.	Phenol, 2,4-bis[1,1-dimethylethyl]	Phenol	Root, Stem and Leaf
2.	Stigmasterol	Phytosterol	Root and Stem
3.	Scandenone (Warangalone)	flavonoid	Root
4.	3,3′-dimethyl-1′-hydroxy-5,8-dimethoxy-2,2′-binapthalene-1,4,5′,8′-tetrone	Napthoquinone derivative	Root and Stem
5.	24,25-dihydroxy vitamin D	Secosteroid	Root
6.	Campesterol	Phytosterol	Stem
7.	ϒ-Sitosterol	Phytosterol	Stem
8.	α-Amyrin	Pentacyclic triterpene	Stem
9.	Cycloartenol acetate	Triterpenoid	Stem
10.	3,7,11,15-Tetramethyl-2-hexadecen-1-ol (phytol)	Terpenoid	Stem
11.	1-Butylpyrrolidine	Alkaloid	Stem
12.	4*H*-Pyran-4-one, 2,3-dihydro-3,5-dihydroxy-6-methyl-	Ketone derivative	Stem
13.	2,3-dihydrobenzofuran (dihydrocoumarone)	Heterocyclic compound	Stem
14.	4-Hydroxy-2-methylacetophenone	Phenol	Stem
15.	2,6-dimethoxy-phenol	Phenol	Stem
16.	4-propoxyphenol	Phenol	Stem
17.	3-Butylindolizidine	Alkaloid	Stem
18.	Pyrogallol	Phenol	Stem
19.	Desulphosinigrin	Glucosinolate	Stem
20.	Lupeol	Pentacyclic triterpenoid	Stem
21.	Dihydrogeraniol	Monoterpenoid	Leaf
22.	α-Caryophyllene (humelene)	Sesquiterpene	Leaf
23.	2(4*H*)-benzofuranone, 5,6,7,7a-terahydro-4,4,7a-trimethyl-(*R*)	Lactone	Leaf
24.	Squalene	Triterpenoid	Leaf

**Table 8:** Effect of ethanolic extract (EE), chloroform extract (CE) and dichloromethane extract (DCME) of *N. triquetra* on carrageenan-induced rat paw edema.

Treatments	Dose of Extract (mg/mL)	Paw Volume (Percentage Inhibition)			
		1 h	2 h	3 h	4 h
Control	Vehicle	--	--	--	--
Indomethacin	10 mg/kg	53.92	57.90	55.41	52.78
Ethanolic Extract (EE)	50 mg/kg	23.94	31.51	29.73	27.78
	100 mg/kg	30.97	35.99	37.84	30.56
	200 mg/kg	38.03	43.84	43.24	31.94
Chloroform Extract (CE)	50 mg/kg	40.84	50.68	48.65	44.44
	100 mg/kg	49.30	54.79	55.41	50.00
	200 mg/kg	49.70	56.26	55.41	52.78
Dichloromethane Extract (DCME)	50 mg/kg	29.58	35.26	33.68	30.66
	100 mg/kg	33.78	39.63	39.29	33.43
	200 mg/kg	35.62	41.37	42.42	37.69

**Table 9:** Effect of ethanolic extract (EE), chloroform extract (CE) and dichloromethane extract (DCME) of *N. triquetra* on cotton pellet granuloma in rats.

Treatment	Dose	Weight of Dry Cotton Pellet (mg)	% Inhibition
Control	Vehicle	81.24 ± 2.63 ^a^	0
Indomethacin	10 mg/kg	35.34 ± 3.23 ^a^	55.39
Ethanolic Extract (EE)	50 mg/kg	65.22 ± 2.50 ^ab^	17.28
	100 mg/kg	50.14 ± 3.47 ^ab^	38.01
	200 mg/kg	48.28 ± 2.66 ^a^	40.46
Chloroform Extract (CE)	50 mg/kg	46.27 ± 2.56 ^a^	43.22
	100 mg/kg	42.27 ± 3.36 ^a^	49.58
	200 mg/kg	40.33 ± 1.66 ^a^	49.87
Dichloromethane Extract (DCME)	50 mg/kg	50.31 ± 3.29 ^ab^	38.26
	100 mg/kg	46.28 ± 2.53 ^a^	42.29
	200 mg/kg	43.67 ± 2.26 ^a^	44.22

The data represent mean value ± SE (standard error), *n* = 6, where *n* = no. of animals in each group, and were found statistically operative and significant by Tukey’s test and the LSD test at *p* < 0.05. Mean ± SE followed by the different letters within each column are significantly different according to Tukey’s test at *p* < 0.05.

**Table 10:** Effect of EE, CE and DCME of *N. triquetra* on exudate volume and neutrophil and monocyte count in carrageenan-induced air-pouch inflammation.

Treatment	Dose	Exudate Volume	Neutrophils (×10 Cells)	Monocytes (×10 Cells)
Control	Vehicle	3.09 ± 0.02 ^a^	219.67 ± 6.64 ^a^	84.50 ± 2.81 ^a^
Indomethacin	10 mg/kg	0.60 ± 0.04 ^a^	69.50 ± 2.49 ^a^	36.67 ± 3.34 ^a^
Ethanolic Extract (EE)	50 mg/kg	2.89 ± 0.08 ^b^	186.67 ± 5.55 ^ab^	71.50 ± 5.78 ^b^
	100 mg/kg	2.25 ± 0.03 ^ab^	156.67 ± 3.01 ^ab^	57.17 ± 2.67 ^ab^
	200 mg/kg	1.55 ± 0.05 ^ab^	120.50 ± 3.91 ^ab^	54.33 ± 2.77 ^a^
Chloroform Extract (CE)	50 mg/kg	1.51 ± 0.03 ^ab^	114.33 ± 3.08 ^ab^	53.03 ± 2.30 ^a^
	100 mg/kg	1.12 ± 0.05 ^ab^	97.83 ± 3.28 ^ab^	43.27 ± 2.26 ^a^
	200 mg/kg	0.82 ± 0.03 ^ab^	90.67 ± 1.78 ^ab^	42.63 ± 2.72 ^a^
Dichloromethane Extract (DCME)	50 mg/kg	2.21 ± 0.04 ^ab^	137.17 ± 4.28 ^ab^	59.37 ± 4.19 ^ab^
	100 mg/kg	1.53 ± 0.03 ^ab^	123.50 ± 3.49 ^ab^	53.60 ± 4.16 ^a^
	200 mg/kg	1.39 ± 0.05 ^ab^	120.17 ± 1.74 ^ab^	52.23 ± 2.27 ^a^

The data represent mean value ± SE (standard error), *n* = 6, where *n* = no. of animals in each group, and were found statistically operative and significant by Tukey’s test and the LSD test at *p* < 0.05. Mean ± SE followed by the different letters within each column are significantly different according to Tukey’s test at *p* < 0.05.

**Table 11:** Effect of EE, CE and DCME of *N. triquetra* on biochemical parameters in serum.

Treatment	Biochemical Parameters					
	AST (IU/L)	ALT (IU/L)	ALP (IU/L)	GGT (IU/L)	Total Protein (mg/DL)	Total Lipids (mg/DL)
**Group I** (Control, LP only)	20.28 ± 0.26 ^a^	25.35 ± 0.37 ^a^	70.35 ± 0.29 ^b^	3.30 ± 0.14 ^a^	5.53 ± 0.14 ^b^	131.33 ± 0.92 ^a^
**Group II** (LP + CCl_4_)	31.25 ± 0.41 ^a^	56.27 ± 0.49^a^	114.59 ± 0.55 ^a^	18.40 ± 0.30 ^a^	3.87 ± 0.25 ^a^	259.17 ± 2.64 ^a^
**Group III** (LP + CCl_4_ + silymarin 25 mg/kg)	21.78 ± 0.16 ^ab^	27.19 ± 0.26 ^b^	74.54 ± 0.59 ^ab^	3.51 ± 0.24 ^b^	5.17 ± 0.31 ^b^	146.20 ± 2.01 ^ab^
**Group IV** (LP + CCl_4_ + EE 50 mg/kg)	24.55 ± 0.18 ^ab^	39.90± 0.63 ^ab^	82.29 ± 0.91 ^ab^	4.72 ± 0.29 ^ab^	5.11± 0.25 ^b^	164.47 ± 5.47 ^ab^
**Group V** (LP + CCl_4_ + EE 100 mg/kg)	22.12 ± 0.31 ^ab^	28.33± 0.23 ^ab^	71.84 ± 0.78 ^b^	3.51 ± 0.18 ^b^	5.39± 0.27 ^b^	146.37 ± 1.11 ^ab^
**Group VI** (LP + CCl_4_ + EE 200 mg/kg)	22.19 ± 0.14 ^ab^	27.83± 0.54 ^ab^	71.71 ± 0.52 ^b^	3.75 ± 0.11 ^b^	5.41± 0.39 ^b^	141.17 ± 1.74 ^b^
**Group VII** (LP + CCl_4_ + CE 50 mg/kg)	21.83 ± 0.27 ^ab^	34.36± 0.29 ^ab^	76.81 ± 0.87 ^ab^	4.30 ± 0.11 ^b^	5.33± 0.21 ^b^	145.12 ± 2.62 ^b^
**Group VIII** (LP + CCl_4_ + CE 100 mg/kg)	20.18 ± 0.32 ^b^	26.06± 0.43 ^b^	68.41 ± 0.65 ^b^	3.06 ± 0.26 ^b^	5.49± 0.12 ^b^	143.50 ± 4.04 ^b^
**Group IX** (LP + CCl_4_ + CE 200 mg/kg)	20.43 ± 0.20 ^b^	24.09± 0.26 ^b^	67.98 ± 0.43 ^b^	3.33 ± 0.12 ^b^	5.67± 0.21 ^b^	135.83 ± 3.17 ^b^
**Group X** (LP + CCl_4_ + DCME 50 mg/kg)	27.78 ± 0.20 ^ab^	52.45± 0.64 ^ab^	104.05 ± 0.40 ^ab^	8.47 ± 0.24 ^ab^	3.83 ± 0.17 ^a^	202.50 ± 1.52 ^ab^
**Group XI** (LP + CCl_4_ + DCME 100 mg/kg)	24.85 ± 0.31 ^ab^	40.40± 0.66 ^ab^	84.06 ± 0.40 ^ab^	4.34 ± 0.21 ^b^	4.80 ± 0.08 ^b^	165.17 ± 4.66 ^ab^
**Group XII** (LP + CCl_4_ + DCME 200 mg/kg)	22.57 ± 0.31 ^ab^	35.40 ± 0.37 ^ab^	75.96 ± 0.82 ^ab^	3.77 ± 0.32 ^b^	5.28 ± 0.17 ^b^	144.47± 2.68 ^b^

The data represent mean value ± SE (standard error), *n* = 6, where *n* = no. of animals in each group, and were found statistically operative and significant by Tukey’s test and the LSD test at *p* < 0.05. Mean ± SE followed by the different letters within each column are significantly different according to Tukey’s test at *p* < 0.05.

**Table 12:** Effect of EE, CE and DCME of *N. triquetra* on biochemical parameters in liver.

Treatment	Parameters			
	Total Protein (g/100 g)	Total Lipids (mg/100 g)	GSH (µmol/g Liver)	Wet Liver Weight (g)
**Group I** (Control, LP only)	8.23 ± 0.18 ^a^	6.74 ± 0.18 ^a^	5.63 ± 0.03 ^b^	3.17 ± 0.136 ^b^
**Group II** (LP + CCl_4_)	5.86 ± 0.15 ^a^	8.07 ± 0.27 ^a^	0.78 ± 0.05 ^a^	5.20 ± 0.10 ^a^
**Group III** (LP + CCl_4_ + silymarin 25 mg/kg)	8.03 ± 0.12 ^b^	6.51 ± 0.23 ^b^	5.17 ± 0.15 ^ab^	3.62 ± 0.06 ^ab^
**Group IV** (LP + CCl_4_ + EE 50 mg/kg)	7.68 ± 0.24 ^b^	6.84 ± 0.18 ^b^	3.40 ± 0.07 ^ab^	4.18 ± 0.08 ^ab^
**Group V** (LP + CCl_4_ + EE 100 mg/kg)	7.96 ± 0.24 ^b^	6.44 ± 0.31 ^b^	4.48 ± 0.11 ^ab^	3.92 ± 0.07 ^ab^
**Group VI** (LP + CCl_4_ + EE 200 mg/kg)	8.25 ± 0.28 ^b^	6.50 ± 0.14 ^b^	5.25 ± 0.08 ^b^	3.80 ± 0.07 ^ab^
**Group VII** (LP + CCl_4_ + CE 50 mg/kg)	7.90 ± 0.18 ^b^	6.50 ± 0.24 ^b^	4.42 ± 0.06 ^ab^	3.78 ± 0.03 ^ab^
**Group VIII** (LP + CCl_4_ + CE 100 mg/kg)	8.00 ± 0.18 ^b^	6.45 ± 0.21 ^b^	5.20 ± 0.06 ^ab^	3.72 ± 0.05 ^ab^
**Group IX** (LP + CCl_4_ + CE 200 mg/kg)	8.13 ± 0.19 ^b^	6.40 ± 0.17 ^b^	5.43 ± 0.08 ^b^	3.61 ± 0.04 ^ab^
**Group X** (LP + CCl_4_ + DCME 50 mg/kg)	6.50 ± 0.22 ^a^	7.75 ± 0.17 ^a^	1.15 ± 0.08 ^a^	4.80 ± 0.04 ^ab^
**Group XI** (LP + CCl_4_ + DCME 100 mg/kg)	6.83 ± 0.17 ^ab^	7.31 ± 0.13 ^b^	2.65 ± 0.08 ^ab^	4.35 ± 0.08 ^ab^
**Group XII** (LP + CCl_4_ + DCME 200 mg/kg)	7.73 ± 0.19 ^b^	7.03 ± 0.14 ^b^	3.93 ± 0.11 ^ab^	4.28 ± 0.09 ^ab^

The data represent mean value ± SE (standard error), *n* = 6, where *n* = no. of animals in each group, and were found statistically operative and significant by Tukey’s test and the LSD test at *p* < 0.05. Mean ± SE followed by the different letters within each column are significantly different according to Tukey’s test at *p* < 0.05.

## Data Availability

All data are available in this manuscript and as Supplementary Materials.

## Supplementary Materials

The following are available online, Figure S1: Chromatogram of Chloroform extract of *Neptunia triquetra* (Vahl) Benth. Roots.; Figure S2: Chromatogram of Dichloromethane extract of *Neptunia triquetra* (Vahl) Benth. Roots.; Figure S3: Chromatogram of Ethanol extract of *Neptunia triquetra* (Vahl) Benth. Roots.; Figure S4: Chromatogram of Chloroform extract of *Neptunia triquetra* (Vahl) Benth. Stem.; Figure S5: Chromatogram of Dichloromethane extract of *Neptunia triquetra* (Vahl) Benth. Stem.; Figure S6: Chromatogram of Ethanol extract of *Neptunia triquetra* (Vahl) Benth. Stem.; Figure S7: Chromatogram of Chloroform extract of *Neptunia triquetra* (Vahl) Benth. Leaves.; Figure S8: Chromatogram of Dichloromethane extract of *Neptunia triquetra* (Vahl) Benth. Leaves.; Figure S9: Chromatogram of Ethanol extract of *Neptunia triquetra* (Vahl) Benth. Leaves.; Table S1: Compounds identified in the Chloroform extract of *Neptunia triquetra* (Vahl) Benth. Roots.; Table S2: Compounds identified in the Dichloromethane extract of *Neptunia triquetra* (Vahl) Benth. Roots.; Table S3: Compounds identified in the Ethanol extract of *Neptunia triquetra* (Vahl) Benth. Roots.; Table S4: Compounds identified in the Chloroform extract of *Neptunia triquetra* (Vahl) Benth. Stem.; Table S5: Compounds identified in the Dichloromethane extract of *Neptunia triquetra* (Vahl) Benth. Stem.; Table S6: Compounds identified in the Ethanol extract of *Neptunia triquetra* (Vahl) Benth. Stem.; Table S7: Compounds identified in the Chloroform extract of *Neptunia triquetra* (Vahl) Benth. Leaves.; Table S8: Compounds identified in the Dichloromethane extract of *Neptunia triquetra* (Vahl) Benth. Leaves.; Table S9: Compounds identified in the Ethanol extract of *Neptunia triquetra* (Vahl) Benth. Leaves.

## Sample Availability

Samples of the compounds are available from the authors Nasir Aziz Wagay, Shah Rafiq and Mohammad Aslam Rather.
