# Supplementary material for: Secondary Metabolite Profiling, Anti-Inflammatory and Hepatoprotective Activity of Neptunia triquetra (Vahl) Benth
Source: Molecules. 2021 Dec 3;26(23):7353. doi: 10.3390/molecules26237353 (PMC8659018; doi:10.3390/molecules26237353)
Supplement: Supplementary file 1 [file molecules-26-07353-s001.zip › molecules-1472297-supplementary.pdf]

Supplementary File S1: Gas Chromatographic Mass spectrometric Studies

Figure S1: Chromatogram of Chloroform extract of *Neptunia triquetra* (Vahl) Benth. Roots

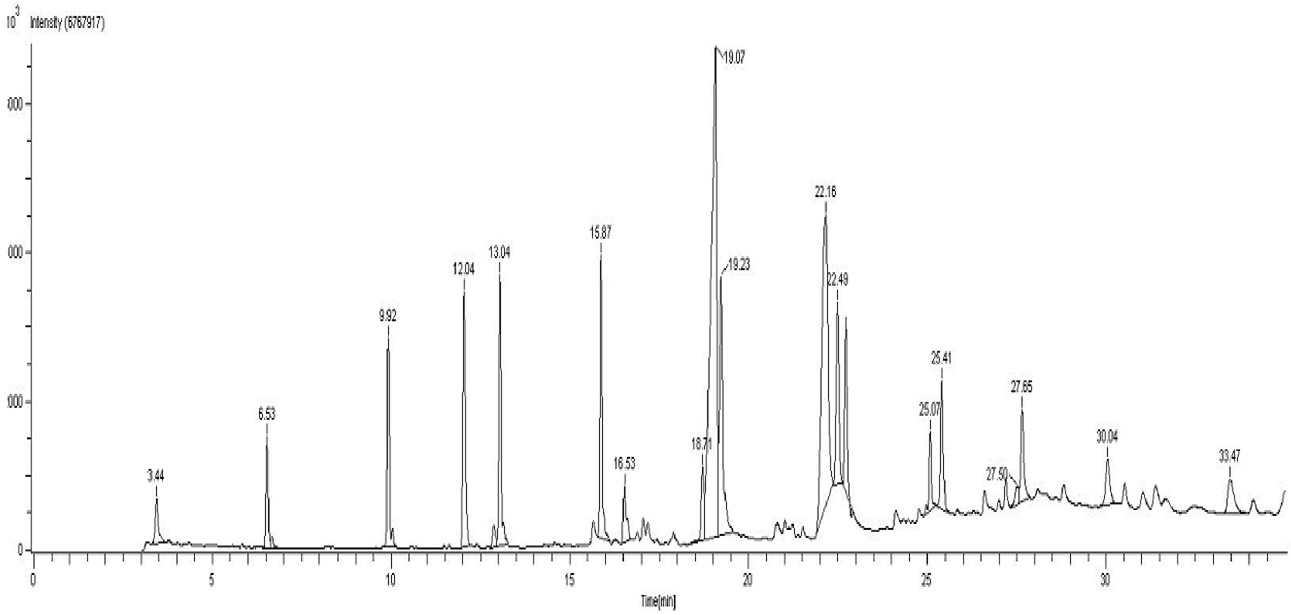

Table S1: Compounds identified in the Chloroform extract of *Neptunia triquetra* (Vahl) Benth. Roots

S. No.	RT	Name of Compound	Peak Area %	MW	MF
1.	9.92	1-Tetradecene	4.19	196	$C_{14}H_{28}$
2	12.04	Phenol 2,4-bis(1,1-dimethylethyl)-	6.08	206	$C_{14}H_{22}O$
3.	13.04	1-Hexadecene	5.61	224	$C_{16}H_{32}$
4.	15.87	3-Octadecene,(E)-	5.72	252	$C_{18}H_{36}$
5.	16.53	3,7,11,15-Tetramethyl-2-hexadecen-1-ol	1.60	296	$C_{20}H_{40}O$
6.	19.07	n-Hexadecanoic acid	25.86	256	$C_{16}H_{32}O_2$
7.	19.23	1-Nonadecene	8.16	266	$C_{19}H_{38}$
8.	22.16	9,12-Octadecadienoic acid (Z.Z)-	16.13	280	$C_{18}H_{32}O_2$
9.	22.49	Octadecanoic acid	4.84	284	$C_{18}H_{36}O_2$
10.	22.71	9-Eicosene, (E)-	3.62	280	$C_{20}H_{40}$
11.	25.07	S-Indacene, 1,2,3,5,6,7-hexahydro-1,1,5,5-tetramethyl-4,8-bis(3-methylbutyl)-	1.80	354	$C_{26}H_{42}$
12.	27.50	3',8,8'-Trimethoxy -3-piperidyl-2,2'-binapthalene-1.1',4,4'-tetrone	0.59	487	$C_{28}H_{25}NO_7$
13.	27.65	Cis-13-Eicosenoic acid	2.64	310	$C_{20}H_{38}O_2$

Figure S2: Chromatogram of Dichloromethane extract of *Neptunia triquetra* (Vahl) Benth. Roots

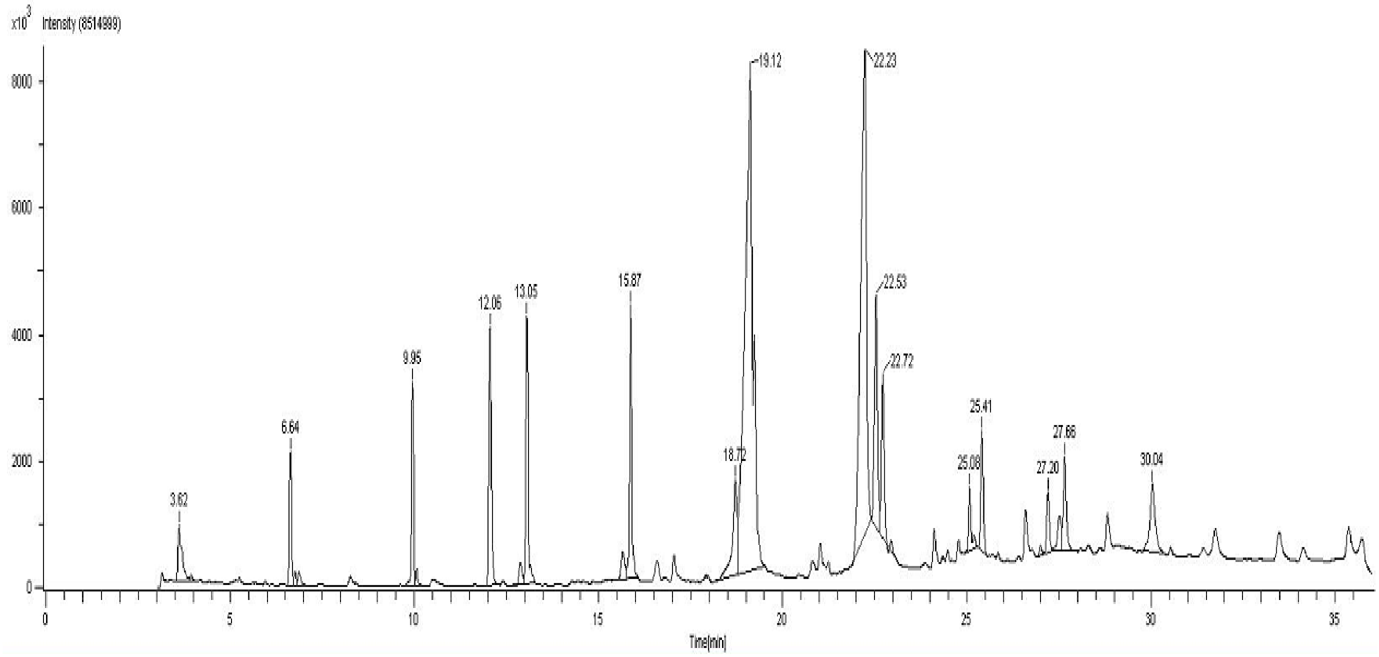

Table S2: Compounds identified in the Dichloromethane extract of *Neptunia triquetra* (Vahl) Benth. Roots.

S. No.	RT	Name of Compound	Peak Area %	MW	MF
1.	3.62	1-Decene	1.71	140	$C_{10}H_{20}$
2.	6.64	1-Dodecene	2.13	168	$C_{12}H_{24}$
3	12.06	Phenol,2,4-bis[1,1-dimethylethyl]-	4.99	206	$C_{14}H_{22}O$
4.	13.05	1-Hexadecene	4.60	224	$C_{16}H_{32}$
5.	15.87	1-Hexadecene	5.01	224	$C_{16}H_{32}$
6.	19.12	n-Hexadecanoic acid	31.21	256	$C_{16}H_{32}O_2$
7.	22.23	Oleic acid	21.59	282	$C_{18}H_{34}O_2$
8.	22.53	Octadecanoic acid	5.38	284	$C_{18}H_{36}O_2$
9.	22.72	1-Docosene	3.26	308	$C_{22}H_{44}$
10.	25.08	S-Indacene, 1,2,3,5,6,7-hexahydro-1,1,5,5-tetramethyl-4,8-bis(3-methylbutyl)-	1.34	354	$C_{26}H_{42}$
11.	25.41	1-Docosene	2.39	308	$C_{22}H_{44}$
12.	27.66	Octatriacontyl pentafluoropropionate	2.19	696	$C_{41}H_{77}F_5O_2$

Figure S3: Chromatogram of Ethanol extract of *Neptunia triquetra* (Vahl) Benth. Roots

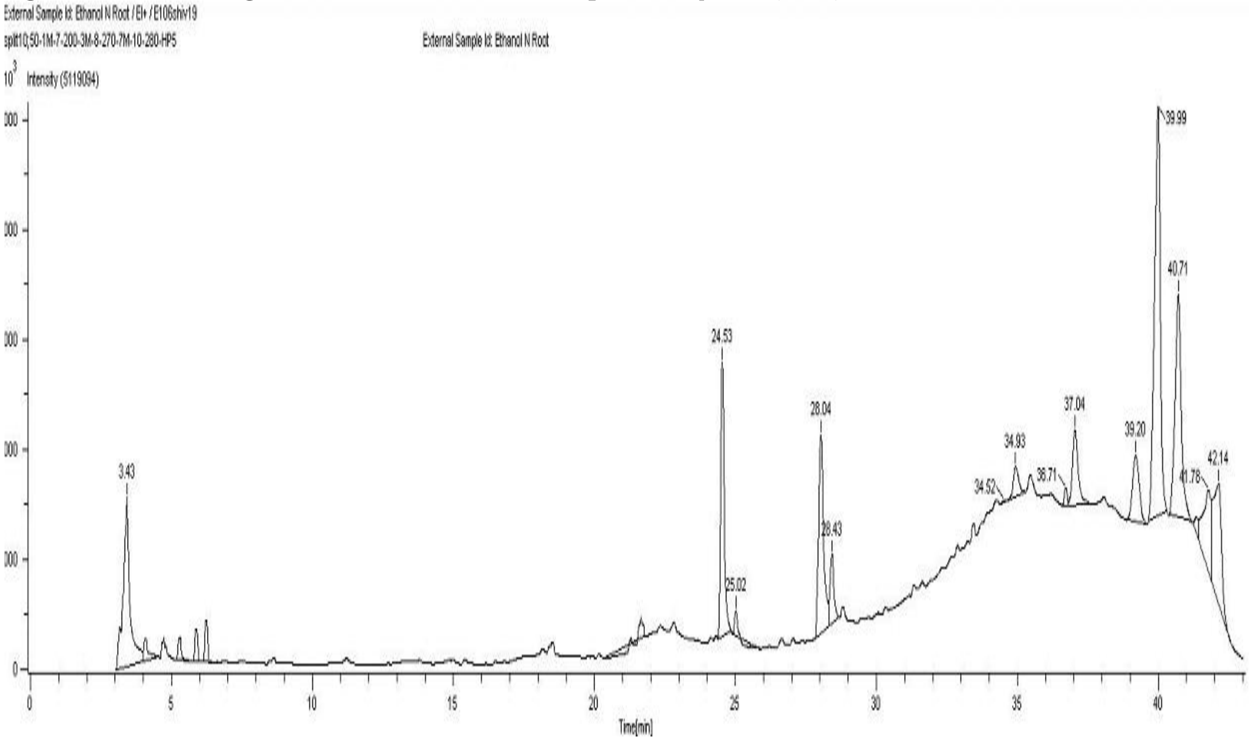

Table S3: Compounds identified in the Ethanol extract of *Neptunia triquetra* (Vahl) Benth. Roots.

S. No.	RT	Name of Compound	Peak Area %	MW	MF
1.	5.31	1,3-Dioxane,4,4-dimethyl-	0.67	116	$C_6H_{12}O_2$
2.	6.25	Butane,1,1-diethoxy-3-methyl-	1.11	160	$C_9H_{20}O_2$
3.	8.62	Propane,1,1,3-triethoxy	0.24	176	$C_9H_{20}O_3$
4.	18.52	Hexadecene	0.46	226	$C_{16}H_{34}$
5.	21.65	1-Hexadecanol,2-methyl-	0.63	256	$C_{17}H_{36}O$
6.	25.02	Hexadecanoic acid,ethyl ester	0.37	284	$C_{18}H_{36}O_2$
7.	28.04	Oleic acid	9.70	282	$C_{18}H_{34}O_2$
8.	37.04	Stigmasterol	4.09	412	$C_{29}H_{48}O$
9.	39.20	Tricyclo[20.8.0.0(7,16)]triacontane,1(22),7(16)-diepoxy-	4.27	444	$C_{30}H_{52}O_2$
10.	39.99	Scandenone	2.29	404	$C_{25}H_{24}O_5$
11.	40.71	3,3’-Dimethyl-1’-hydroxy-5,8-dimethoxy-2,2’-binapthalene-1,4,5’,8’-tetrone	13.78	418	$C_{24}H_{18}O_7$
12.	42.14	24,25-Dihydroxyvitamin D	9.28	416	$C_{27}H_{44}O_3$

Figure S4: Chromatogram of Chloroform extract of *Neptunia triquetra* (Vahl) Benth. Stem

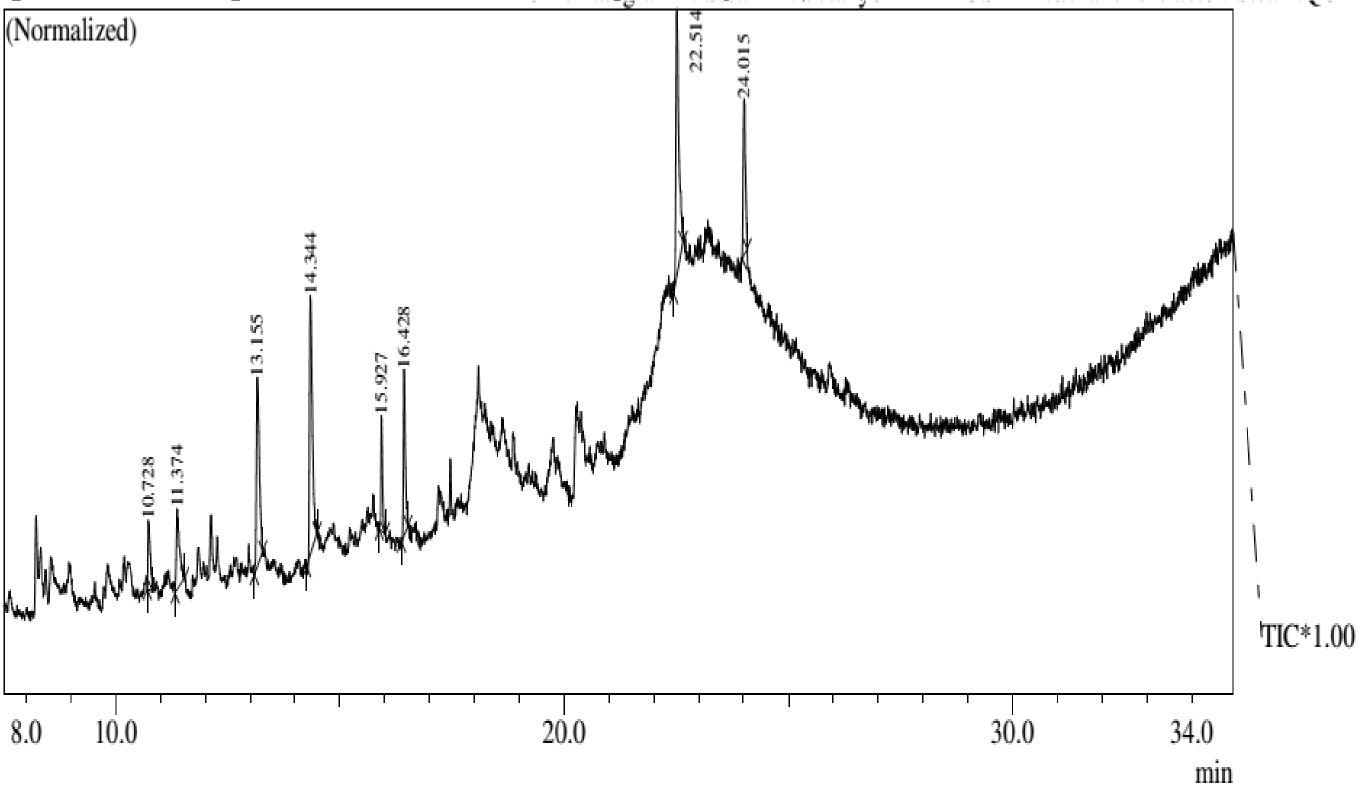

Table S4: Compounds identified in the Chloroform extract of *Neptunia triquetra* (Vahl) Benth. Stem.

S. No.	RT	Name of Compound	Peak Area %	MW	MF
1.	10.728	1-Butylpyrrolidine	4.95	127	$C_8H_{17}N$
2.	11.374	2,4,5-Trihydroxypyrimidine	7.50	128	$C_6H_8O_3$
3.	13.155	4H-Pyran-4-one, 2,3-dihydro-3,5-dihydroxy-6-methyl-	15.73	144	$C_6H_8O_4$
4.	14.344	2,3-dihydrobenzofuran (Dihydrocoumarone)	21.99	120	C_8H_8O
5.	15.927	4-Hydroxy-2-methylacetophenone	5.80	150	$C_9H_{10}O_2$
6.	16.428	Phenol, 2,6-dimethoxy-	9.19	154	$C_8H_{10}O_3$
7.	22.514	Phenol, 4-propoxy-	24.65	152	$C_9H_{12}O_2$
8.	24.015	3-Butylindolizidine	10.20	181	$C_{12}H_{23}N$

Figure S5: Chromatogram of Dichloromethane extract of *Neptunia triquetra* (Vahl) Benth. Stem

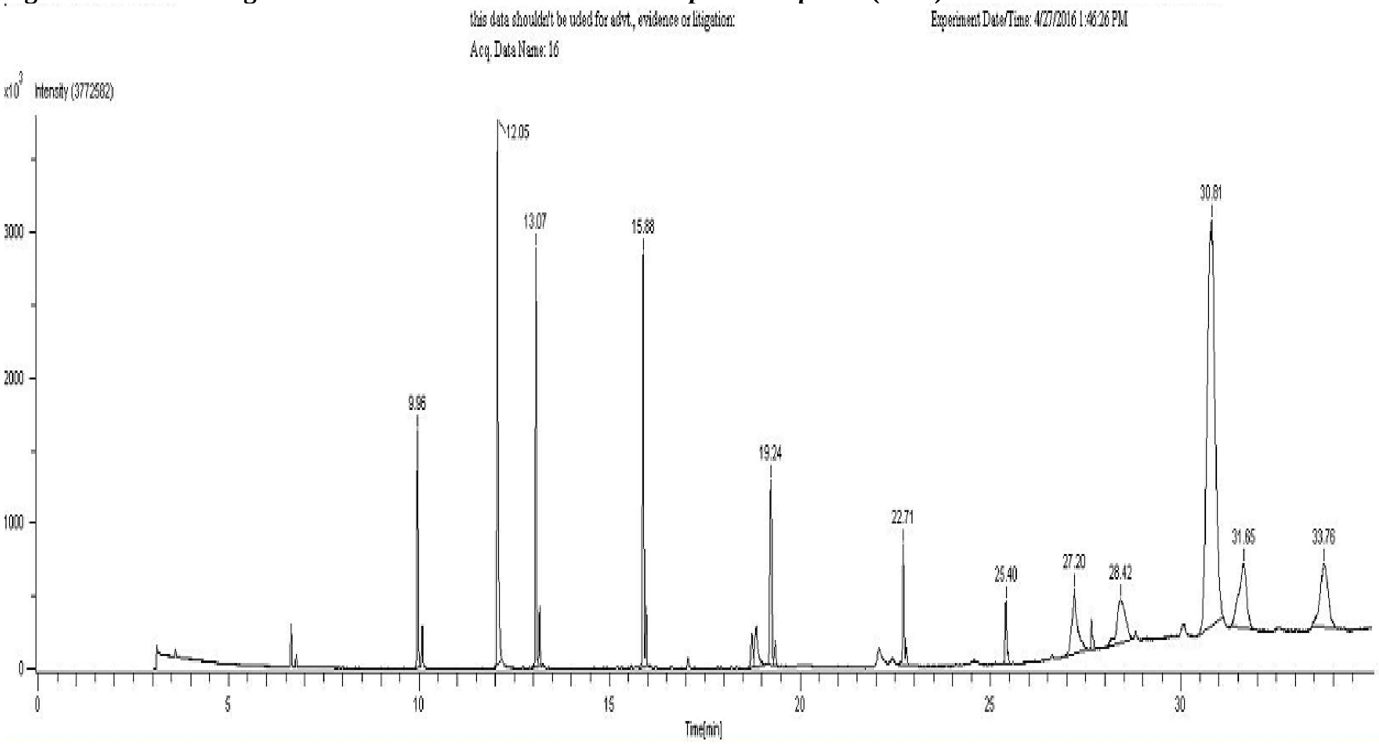

Table S5: Compounds identified in the Dichloromethane extract of *Neptunia triquetra* (Vahl) Benth. Stem

S. No.	RT	Name of Compound	Peak Area %	MW	MF
1.	12.05	Phenol,2,4-bis[1,1-dimethylethyl]-	9.80	206	$C_{14}H_{22}O$
2.	15.88	1-Nonadecene	6.74	266	$C_{19}H_{38}$
3.	18.85	n-Hexadecanoic acid	1.56	256	$C_{16}H_{32}O_2$
4.	27.20	Campesterol	4.30	400	$C_{28}H_{48}O$
5.	28.42	Stigmasterol	4.80	412	$C_{29}H_{48}O$
6.	30.81	ϒ-Sitosterol	40.15	414	$C_{29}H_{50}O$
7.	31.65	α-Amyrin	6.60	426	$C_{30}H_{50}O$
8.	33.76	Cycloartenol acetate	6.45	468	$C_{32}H_{52}O_2$

Figure S6: Chromatogram of Ethanol extract of *Neptunia triquetra* (Vahl) Benth. Stem

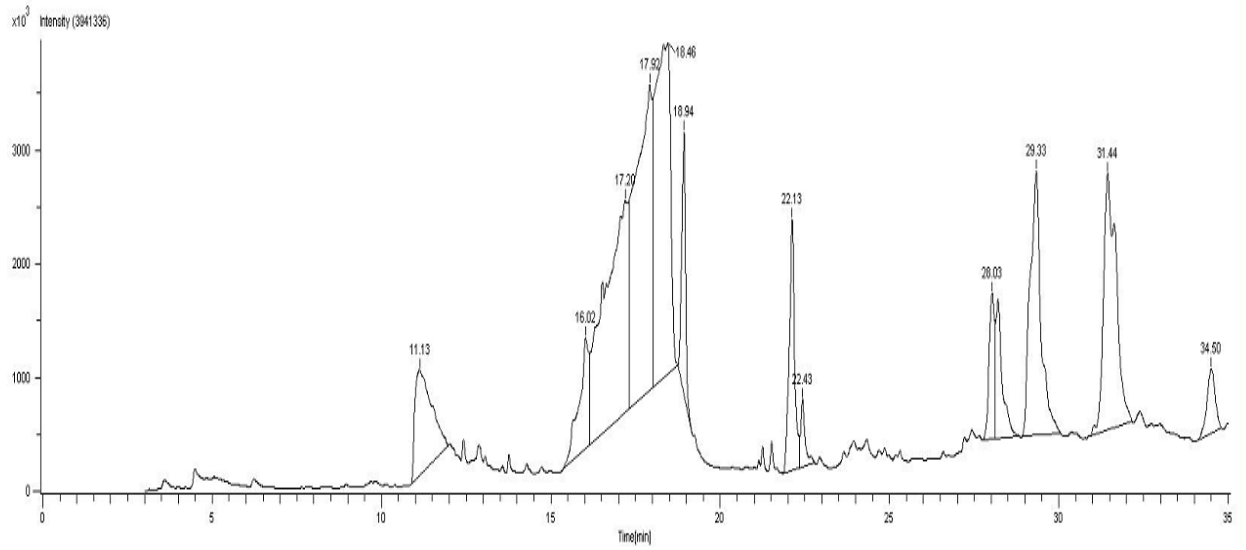

Table S6: Compounds identified in the Ethanol extract of *Neptunia triquetra* (Vahl) Benth. stem

S. No.	RT	Name of Compound	Peak Area %	MW	MF
1.	4.50	1,2,3-Benzenetriol	0.76	126	$C_6H_6O_3$
2.	11.13	Pyrogallol	5.94	126	$C_6H_6O_3$
3.	16.02	Desulphosinigrin	3.71	279	$C_{10}H_{17}NO_6S$
4.	17.20	3-O-Methyl-d-glucose	17.72	194	$C_7H_{14}O_6$
5.	18.46	3-O-Methyl-d-glucose	17.08	194	$C_7H_{14}O_6$
6.	18.94	n-Hexadecanoic acid	3.46	256	$C_{16}H_{32}O_2$
7.	21.52	Phytol	0.24	296	$C_{20}H_{40}O$
8.	22.13	Linolenic acid	4.49	278	$C_{18}H_{30}O_2$
9.	22.43	Octadecanoic acid	1.01	284	$C_{18}H_{36}O_2$
10.	29.33	Stigmasterol	9.80	412	$C_{29}H_{48}O$
11.	31.44	3,3’-Dimethyl-1’-hydroxy-5,8-dimethoxy-2,2’-binapthalene-1,4,5’,8’-tetrone	11.09	418	$C_{24}H_{18}O_7$
12	31.64	ϒ-Sitosterol	8.88	414	$C_{29}H_{50}O$
13.	34.50	Lupeol	1.80	426	$C_{30}H_{50}O$

Figure S7: Chromatogram of Chloroform extract of *Neptunia triquetra* (Vahl) Benth. Leaves

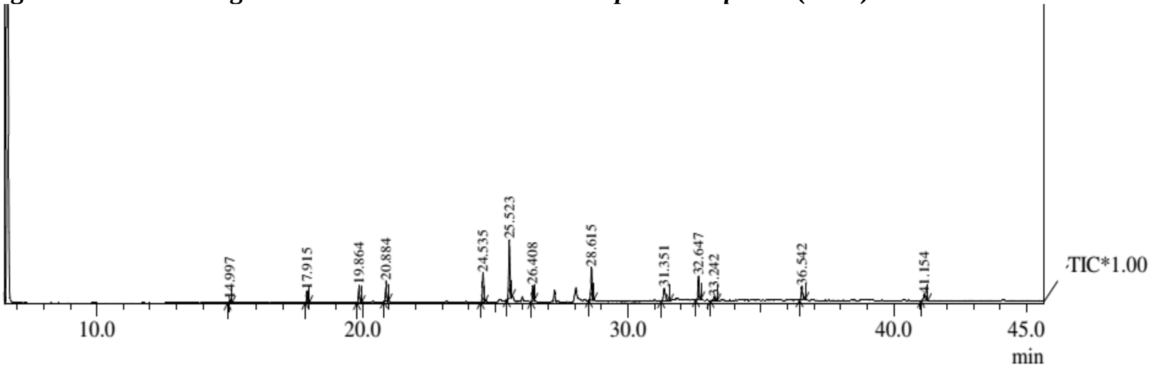

Table S7: Compounds identified in the Chloroform extract of *Neptunia triquetra* (Vahl) Benth. Leaves

S. No.	RT	Name of Compound	Peak Area %	MW	MF
1.	14.997	1-Dodecanol	1.02	186	$C_{12}H_{26}O$
2.	17.915	1-Tetradecano	3.88	214	$C_{14}H_{30}O$
3.	19.864	Phenol, 3,5-bis(1,1-dimethylethyl)-	7.03	206	$C_{14}H_{22}O$
4.	20.884	1-Pentadecene	6.64	210	$C_{15}H_{30}$
5.	24.535	1-Octadecanol	11.56	270	$C_{18}H_{38}O$
6.	25.523	3,7,11,15-Tetramethyl-2-hexadecen-1-ol	22.27	296	$C_{20}H_{40}O$
7.	26.408	3,7,11,15-Tetramethyl-2-hexadecen-1-ol	5.98	296	$C_{20}H_{40}O$
8.	28.615	1-Heptadecanol	12.40	256	$C_{17}H_{36}O$
9.	31.351	Dihydrogeraniol	8.74	156	$C_{10}H_{20}O$
10.	32.647	Behenic alcohol	8.76	326	$C_{22}H_{46}O$
11.	33.242	Bicyclo[3.1.1]heptan-3-one, 2,6,6-trimethyl-	1.83	152	$C_{10}H_{16}O$
12.	36.542	1-Nonadecene	6.14	266	$C_{19}H_{38}$
13.	41.154	1-Heneicosyl formate	3.76	340	$C_{22}H_{44}O_2$

Figure S8: Chromatogram of Dichloromethane extract of *Neptunia triquetra* (Vahl) Benth. Leaves

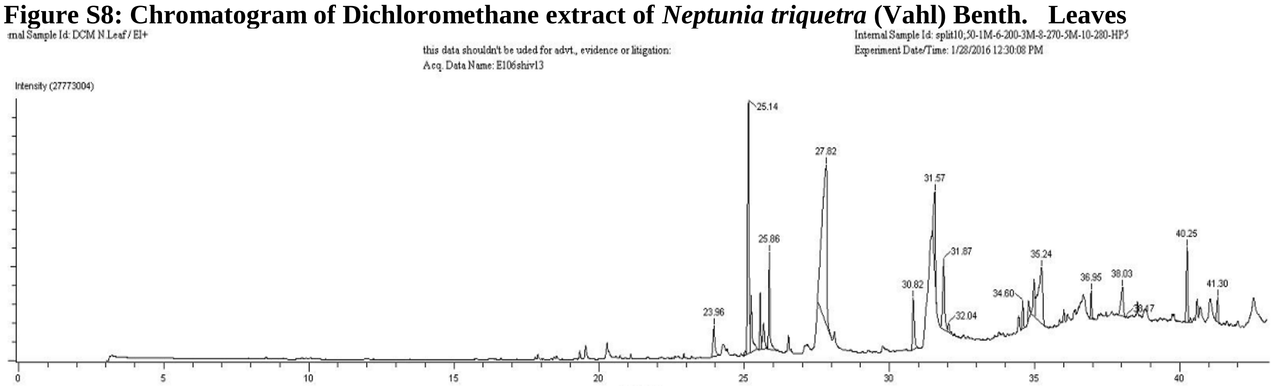

Table S8: Compounds identified in the Dichloromethane extract of *Neptunia triquetra* (Vahl) Benth. Leaves.

S. No.	RT	Name of Compound	Peak Area %	MW	MF
1.	17.89	Humelene	0.46	204	$C_{15}H_{24}$
2.	19.53	2(4H)-Benzofuranone, 5,6,7,7a-terahydro-4,4,7a-trimethyl-(R)-	0.76	180	$C_{11}H_{16}O_2$
3.	20.28	Dodecanoic acid	1.00	200	$C_{12}H_{24}O_2$
4.	23.95	Tetradecanoic acid	1.28	228	$C_{14}H_{28}O_2$
5.	25.14	3,7,11,15-Tetramethyl-2-hexadecen-1-ol	10.10	296	$C_{20}H_{40}O$
6.	25.24	2-Pentadecanone, 6,10,14-trimethyl-	1.86	268	$C_{18}H_{36}O$
7.	25.56	3,7,11,15-Tetramethyl-2-hexadecen-1-ol	2.37	296	$C_{20}H_{40}O$
8.	27.80	n-Hexadecanoic acid	26.59	256	$C_{16}H_{32}O_2$
9.	30.83	Phytol	2.35	296	$C_{20}H_{40}O$
10.	31.54	Linolenic acid	18.79	278	$C_{18}H_{30}O_2$
11.	31.86	Octadecanoic acid	3.28	284	$C_{18}H_{36}O_2$
12.	38.02	Octadecane, 3-ethyl-5-(2-ethylbutyl)	2.02	366	$C_{26}H_{54}$
13.	40.60	Squalene	1.58	410	$C_{30}H_{50}$
14.	42.55	Octadecane, 3-ethyl-5-(2-ethylbutyl)	3.25	366	$C_{26}H_{54}$

Figure S9: Chromatogram of Ethanol extract of *Neptunia triquetra* (Vahl) Benth. Leaves

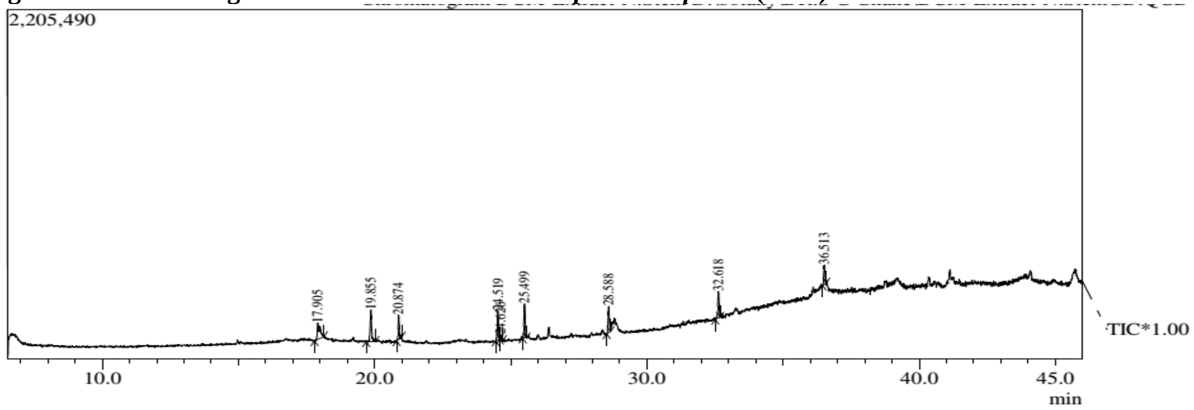

Table S9: Compounds identified in the Ethanol extract of *Neptunia triquetra* (Vahl) Benth. Leaves.

S. No.	RT	Name of Compound	Peak Area %	MW	MF
1.	17.905	1-Heptadecene	14.60	238	$C_{17}H_{34}$
2.	19.855	Phenol, 2,4-bis(1,1-dimethylethyl)-	15.91	206	$C_{14}H_{22}O$
3.	20.874	1-Heptadecanol	8.95	256	$C_{17}H_{36}O$
4.	24.519	1-Octadecanol	12.39	270	$C_{18}H_{38}O$
5.	24.626	Norphytan	1.65	268	$C_{19}H_{40}$
6.	25.499	3,7,11,15-Tetramethyl-2-hexadecen-1-ol	15.14	296	$C_{20}H_{40}O$
7.	28.588	1-Nonadecene	11.23	266	$C_{19}H_{38}$
8.	32.618	Behenic alcohol	12.01	326	$C_{22}H_{46}O$
9.	36.513	1-Eicosanol	8.10	298	$C_{20}H_{42}O$

Molecular structures of Identified Secondary Metabolites

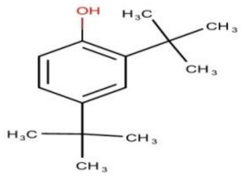

Phenol,2,4-bis[1,1-dimethylethyl]-

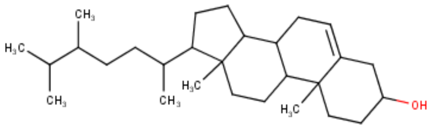

Campesterol

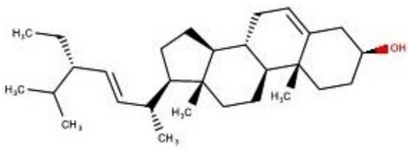

Stigmasterol

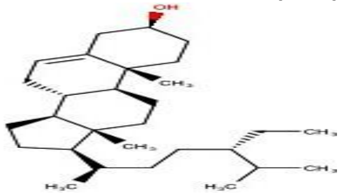

ϒ-Sitosterol

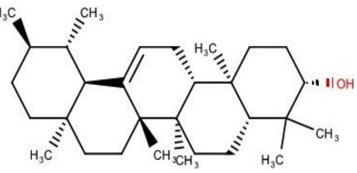

α-Amyrin

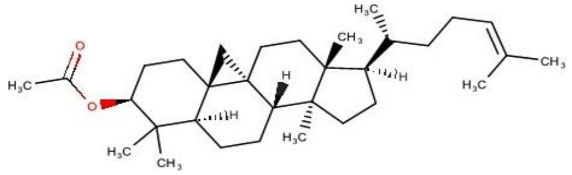

Cycloartenol acetate

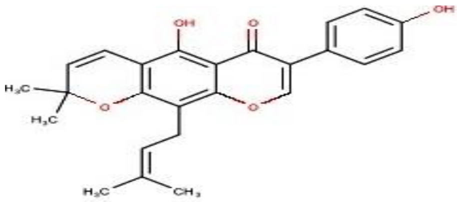

Scandenone (Warangalone)

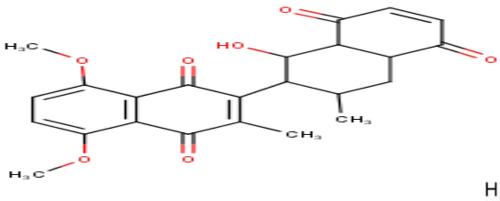

3,3’-Dimethyl-1’-hydroxy-5,8-dimethoxy-2,2’-binapthalene-1,4,5’,8’-tetrone

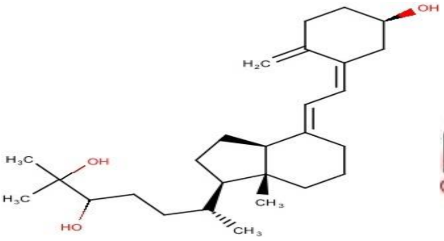

24,25-Dihydroxyvitamin D

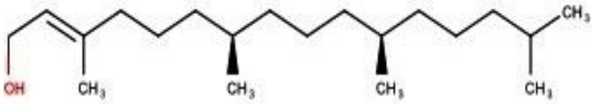

3,7,11,15-Tetramethyl-2-hexadecen-1-ol

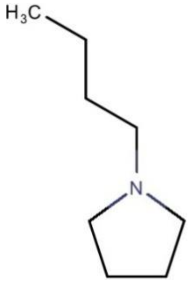

1-Butylpyrrolidine

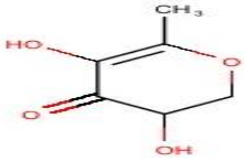

4H-Pyran-4-one, 2,3-dihydro, -3,5-dihydroxy-6-methyl

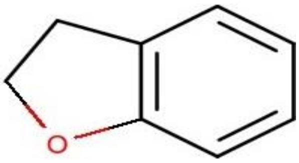

Benzofuran, 2,3-dihydro-

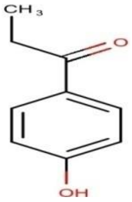

4-Hydroxy-2-methylacetophenone

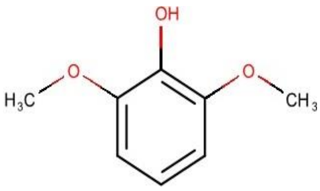

Phenol, 2,6-dimethoxy-

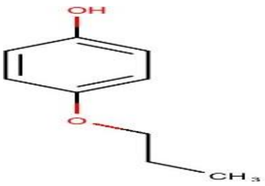

Phenol, 4-propoxy-

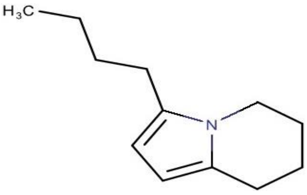

3-Butylindolizidine

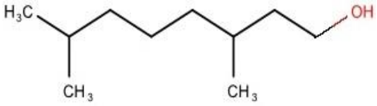

Dihydrogeraniol

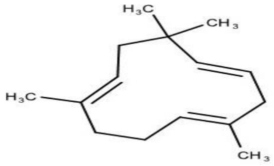

α-Caryophyllene (Humelene)

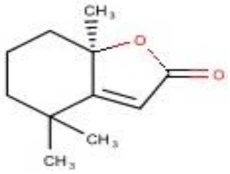

2(4H)-Benzofuranone 5,6,7,7a-terahydro-4,4,7a-trimethyl-(R)-

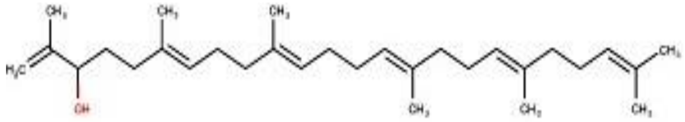

Squalene

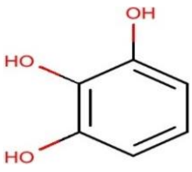

Pyrogallol

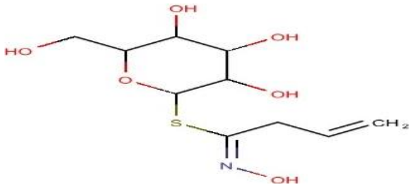

Desulphosinigrin

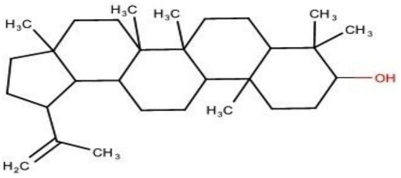

Lupeol
